# Involvement of the Substance P/Neurokinin-1 Receptor System in Cancer

**DOI:** 10.3390/cancers14143539

**Published:** 2022-07-21

**Authors:** Rafael Coveñas, Miguel Muñoz

**Affiliations:** 1 Laboratory of Neuroanatomy of the Peptidergic Systems, Institute of Neurosciences of Castilla and León (INCYL), University of Salamanca, 37007 Salamanca, Spain; 2 Group GIR-USAL: BMD (Bases Moleculares del Desarrollo), University of Salamanca, 37007 Salamanca, Spain; 3 Pediatric Intensive Care Unit, Virgen del Rocío University Hospital, Avda. Manuel Siurot s/n, 41013 Seville, Spain; 4 Research Laboratory on Neuropeptides, Institute of Biomedicine of Seville (IBIS), 41013 Seville, Spain

New, promising molecular targets to block tumor development and new compounds capable of specifically destroying cancer cells must be urgently investigated. In recent years, the knowledge on the involvement of the substance P (SP)/neurokinin-1 receptor (NK-1R) system in cancer has attracted increasing interest [1,2,3,4,5]. This has opened up new research lines and possibilities to improve cancer diagnosis and to explore new therapeutic strategies targeting tumor-specific molecular derangements since SP/NK-1R expression has been associated with cancer development and poor prognosis [1,2,3,4,5]. SP, an undecapeptide that binds preferentially to NK-1R, plays crucial roles in cancer development [1,2,3,4,5,6]: (1) it promotes the mitogenesis/migration of tumor cells; (2) it exerts antiapoptotic action; (3) it increases the glycolytic rate (Warburg effect); (4) it stimulates the growth of blood vessels, and (5) tumor cells also synthesize and release SP, which acts through autocrine, paracrine, and neuroendocrine (tumor mass) mechanisms. Moreover, tumor cells also overexpress NK-1R, which is involved in their viability; however, it has recently been demonstrated that this receptor is not essential for the viability of normal cells [7]. This is extremely important since the overexpression of NK-1R opens up the possibility for a specific therapeutic treatment against cancer cells using NK-1R antagonists (e.g., the drug aprepitant, which exerts a broad-spectrum anticancer effect) [8] and, in addition, the overexpression of NK-1R could be used as a prognostic biomarker. In fact, NK-1R antagonists promote apoptosis in tumor cells in a concentration-dependent manner, block the migration of cancer cells, prevent metastasis, and inhibit angiogenesis; that is, NK-1R antagonists act as broad-spectrum antineoplastic drugs against tumor cells overexpressing NK-1R, which occurs in many types of cancers [6]. Chemotherapy/radiotherapy possess significant limitations: chemoresistance and severe side-effects. In combination therapy with chemotherapy/radiotherapy, NK-1R antagonists (e.g., aprepitant) exert a synergic effect, promote tumor chemosensitization/radiosensitization, and counteract the severe side-effects (e.g., nephrotoxicity, cardiotoxicity, neurotoxicity) promoted by cytostatics [9]. In fact, the combined therapy of aprepitant with doxorubicin exerted the chemosensitization of cancer cells and decreased the cardiotoxicity promoted by the cytostatic effect on cardiomyocytes [10]. Because cancer cells overexpress NK-1R, this combination strategy is a promising approach for the treatment of many kinds of cancer and might open the door to a new era in chemotherapy/radiotherapy; this combination therapy could translate into better quality of life, higher cure rates, and fewer sequelae in cancer patients [9]. Importantly, the absence of the stimulus mediated by SP using NK-1R inhibitors (genetic or pharmacological treatment with NK-1R antagonists) induces the death of tumor cells by apoptosis [6]; this crucial finding and previous observations mean that NK-1R is a potential new target in cancer treatment and that NK-1R antagonists represent a promising generation of new antitumor drugs.

The main goal of this Special Issue is to increase the knowledge about the multiple roles played by the SP/NK-1R system in cancer development and to show evidence of the promising use of NK-1R antagonists as a new antitumor strategy; the researchers participating in the Special Issue have fully achieved this goal. They have focused their research on the following key points [11,12,13,14,15]: (1) a novel potential therapeutic approach to treat triple negative breast cancer cells, (2) the use of aprepitant as a tool for preventing chemotherapy-associated cardiotoxicity, (3) the involvement of the SP/NK-1R system in head-and-neck carcinogenesis, (4) the use of NK-1R antagonists as a new strategy to overcome cancer resistance, and (5) the importance of NK-1R as a targetable stratification factor for drug repurposing in pancreatic cancer. It is known that cisplatin is effective against triple-negative breast cancer (TNBC) cells; however, it has unwarranted outcomes owing to neurotoxicity, chemoresistance, and recurrence [11]. In this Special Issue, Dr. Prema Robinson and co-workers from the University of Texas MD Anderson Cancer Center showed, in vitro, a novel therapeutic option to increase the efficacy of cisplatin in TNBC and, in addition, they demonstrated that this therapeutic option also protected neuronal cells from cisplatin-induced toxicity [11]. In this study, Dr. Robinson et al. [11] observed a high NK-1R level in response to cisplatin in neuronal and TNBC cell lines, whereas treatment with the NK-1R antagonist aprepitant decreased cisplatin-induced apoptosis, reactive oxygen species production, and viability loss in neuronal cells; however, in TNBC cells, an increase was observed. Importantly, Dr. Robinson and co-workers also showed that genes associated with cell-cycle progression, chemoresistance, inflammation, and metastases were attenuated by SP-receptor antagonism in one of the TNBC cell lines studied (Sum 185) [11]. This study suggests that the use of aprepitant in combination therapy with cisplatin is a safer and more efficacious therapeutic option than the current therapies used against TNBC cells. In sum, this is a novel potential therapeutic approach to treat TNBC cells. In a second paper, the same group studied the cardiotoxicity induced by the administration of doxorubicin (DOX) [12]. This compound is an anthracycline broadly used to treat both solid and blood tumors; however, due to its cardiotoxicity, the clinical use of DOX is limited. Here, Dr. Robinson et al. [12] studied this limitation and demonstrated that aprepitant prevented chemotherapy-induced cardiotoxicity in vivo. The authors observed interesting findings: (1) SP/NK-1R levels increased in the heart of animals treated with DOX; (2) in these animals, aprepitant decreased the levels of cardiomyocyte hypertrophy, oxidative stress, and apoptosis; and (3) in DOX-treated animals, aprepitant decreased the actions mediated by DOX on fractional shortening, ejection fraction, and stroke volume to values not significantly different from sham animals The main finding reported by Robinson et al. [12] is that the SP/NK-1R system mediates cardiomyocyte hypertrophy and cardiac oxidative stress, apoptosis, and dysfunction and that aprepitant is a useful tool for preventing chemotherapy-associated cardiotoxicity in cancer. Dr. Miguel Ángel González-Moles and his group from the University of Granada and WHO Collaborating Group for Oral Cancer performed a systematic review and meta-analysis to show the involvement of the SP/NK-1R system in head-and-neck carcinogenesis [13]. This systematic review is important since about 600,000 new cases/year of head-and-neck cancer are diagnosed and around 300,000 deaths/year are reported [13]. Thanks to this systematic review and meta-analysis, the authors reported that the upregulation of the SP/NK-1R system is an oncogenic event involved in head-and-neck carcinogenesis by acting in the early stages of malignization. Moreover, the authors highlighted a greater relevance of the upregulation of NK-1R compared to SP, showing the important role that this receptor will play in the development of new targeted therapies [13]. The SP/NK-1R system is also involved in molecular pathways associated with cancer resistance. Dr. Maximino Redondo and his group from the University of Málaga reviewed the use of NK-1R antagonists as a new strategy to overcome cancer resistance; they suggest that the repurposing of aprepitant, which is a safe and antiemetic marketed NK-1R antagonist, will help to overcome this resistance [14]. This is an important point since the resistance shown by tumor cells to anticancer drugs is one of the main causes of anticancer treatment failures and deaths; in fact, drug resistance is responsible for over 90% of deaths observed in patients treated with new anticancer treatments or chemotherapy [16]. Redondo et al. [14] state that studies focused on the use of NK-1R antagonists (monotherapy or combination therapy) for the treatment of resistant tumors must be carried out in the future. Finally, Dr. Matthias Ilmer and his group from the Ludwig Maximilians University of Munich showed that human pancreatic ductal adenocarcinoma (PDAC) cells express NK-1R, that aprepitant decreases cell growth, and that this drug does not promote apoptosis but induces the arrest of the cell cycle [15]. This is another important point to study in-depth since the most common type of pancreatic cancer is PDAC, which shows very limited therapeutic options and a low life expectancy. Dr. Ilmer and co-workers reported that the main NK-1R isoform located in PDAC cells was the truncated form and that the highest antitumor action exerted by aprepitant was observed in aggressive tumor cells expressing higher levels of the truncated NK-1R. Gene expression and key genes in PDAC tumorigenesis were also analyzed; in patients showing a high level of NK-1R, a better overall survival was observed and, at the same time, the expression of NK-1R was decreased in PDAC tissues when it was compared to normal tissues. In the transcriptomic records consulted by the authors, no differentiation between isoforms (full, truncated) of NK-1R was found [15]. The authors concluded that the analysis of splice variants could lead to the stratification of PDAC patients for NK-1R-directed therapeutic strategies and that this receptor could be important in future personalized medicine. 

Currently, the main findings regarding the involvement of the SP/NK-1R system in cancer are the following [6,8]: (1) SP is a mitogenic agent in many types of cancer cells, (2) NK-1R antagonists also exert an antitumor action, and (3) NK-1R is an antitumor therapeutic target because cancer cells overexpress the receptor. Why do tumor cells overexpress NK-1R? Although many of the molecular mechanisms involved in this process are currently unknown, the answer is quite simple: because the stimulus mediated by SP, after binding to NK-1R, is beneficial for both the survival of tumor cells (proliferation, migration, antiapoptotic, and Warburg effects) and tumor development (angiogenesis); hence, tumor cells enhance the expression of NK-1R by currently unknown mechanisms. That is, tumor cells ensure the binding of SP, which come from multiple sources: the peptide can be released from the tumor cell itself, from immune cells located in the tumor microenvironment, and from nerve terminals; in addition, circulating SP can reach cancer cells from the bloodstream [6]. In the case that tumor cells do not receive the beneficial stimulus mediated by SP (blocking with NK-1R antagonists or inhibiting the synthesis of NK-1R), apoptotic mechanisms are activated in cancer cells [6,8]. Accordingly, what are the most important scientific challenges in the near future regarding the SP/NK-1R system and cancer? In this Special Issue and in previous in vitro and in vivo studies, the use of aprepitant as an antitumor agent has fully been demonstrated [1,2,3,6,8,9,12,14,15]. This antiemetic drug is a perfect candidate to be used as an antitumor drug, and for this reason, the repurposing of this drug alone or in combination therapy is urgently required: this is the main scientific challenge. In general, the IC_100_ of aprepitant for cancer cells is 50 µM and the IC_50_ and IC_100_ for non-tumor cells are 60 µM and 90 µM, respectively [6]. The safety of aprepitant is excellent; it is well tolerated, and it has recently been suggested that the clinical antitumor dose of aprepitant to be administered could be 20–40 mg/kg/day and that, according to the response of the treatment, the days of administration would have to be increased compared to the three days that it is currently administered in clinical practice [6]. Thus, phase I and II clinical trials are needed to assess the safety and efficacy of aprepitant at a higher dose than that currently used in clinical practice as antiemetic. What if it was simply a question of dosage [17]? The knowledge on the involvement of SP/NK-1R in cancer has increased in recent years thanks to a few researchers and laboratories around the world [1,2,3,4,5,6,7,8,9,10,11,12,13,14,15,17,18,19,20,21,22]; the findings reported by these researchers greatly support the initiation of clinical studies to confirm the antitumor action of aprepitant. We hope that this Special Issue will serve to increase the scientific interest for the SP/NK-1R system, not only among basic researchers but also in the pharmaceutical industry, to join forces to fight cancer. Knowing the crucial involvement of the SP/NK-1R system in cancer, demonstrated in many previous in vitro and in vivo works, and seeing what has been shown in this Special Issue, two crucial exciting questions arise: are we currently in the right direction to fight cancer? What are we waiting for to increase the knowledge on the roles played by the SP/NK-1 system in cancer from a preclinical and clinical point of view? This Special Issue has fully contributed to increase this knowledge.
